# *Drosophila* larval epidermal cells only exhibit epidermal aging when they persist to the adult stage

**DOI:** 10.1242/jeb.240986

**Published:** 2021-05-06

**Authors:** Yan Wang, Sirisha Burra, Michael J. Galko

**Affiliations:** 1 Department of Genetics, University of Texas MD Anderson Cancer Center, Houston, TX 77030, USA; 2 Genetics & Epigenetics Graduate Program, MD Anderson UTHealth Graduate School of Biomedical Sciences, Houston, TX 77030, USA

**Keywords:** Adult epidermal cells, Aging signals, Larvae, Heterochronic, *Drosophila*

## Abstract

Holometabolous insects undergo a complete transformation of the body plan from the larval to the adult stage. In *Drosophila*, this transformation includes replacement of larval epidermal cells (LECs) by adult epidermal cells (AECs). AECs in *Drosophila* undergo a rapid and stereotyped aging program where they lose both cell membranes and nuclei. Whether LECs are capable of undergoing aging in a manner similar to AECs remains unknown. Here, we addressed this question in two ways. First, we looked for hallmarks of epidermal aging in larvae that have a greatly extended third instar and/or carry mutations that would alter the pace of epidermal aging at the adult stage. Such larvae, irrespective of genotype, did not show any of the signs of epidermal aging observed in the adult. Second, we developed a procedure to effect a heterochronic persistence of LECs into the adult epidermal sheet. Lineage tracing verified that presumptive LECs in the adult epidermis are not derived from imaginal epidermal histoblasts. LECs embedded within the adult epidermal sheet undergo clear signs of epidermal aging; they form multinucleate cells with each other and with the surrounding AECs. The incidence of adult cells with mixed AEC nuclei (small) and persistent LEC nuclei (large) increased with age. Our data reveal that epidermal aging in holometabolous *Drosophila* is a stage-specific phenomenon and that the capacity of LECs to respond to aging signals does exist.

## Abstract (precis)

**Summary:** *Drosophila* larval epidermal cells do not age at the larval stage; however, they do exhibit signs of aging if they persist into the adult stage.

## INTRODUCTION

Holometabolous insects undergo a complete restructuring of the body plan during metamorphosis. Typically, this involves changing the morphology of a larva (usually a worm-like transitional stage) into an adult that possesses adult appendages (legs, wings, antennae) and functional reproductive organs. In some insects, including Drosophilid flies, the larval stage can enter a diapause which temporarily halts further development (Enomoto, 1981) until the local environment is conducive to further growth. In a phenomenon distinct from diapause, certain dietary restrictions that prevent synthesis of the molting hormone (Parkin and Burnet, 1986) can block the onset of pupariation and result in long-lived *Drosophila* larvae. A variety of genetic mutations also result in a greatly prolonged larval stage (Belinski-Deutsch et al., 1983; Sandoval et al., 2014), often with no pupariation or metamorphosis.

As in most insects, the *Drosophila* larval epidermis is a monolayer of large polarized polygonal epithelial cells that are adherent to an apical cuticle (Gangishetti et al., 2012) and that synthesize a basal lamina (Fessler and Fessler, 1989) separating them from the hemolymph in the open body cavity. Little is known about whether or how prolonged larval stages affect barrier tissue architecture. During metamorphosis, epidermal histoblast cells (Madhavan and Madhavan, 1980) proliferate and migrate to replace the larval epidermal cells (LECs) undergoing apoptosis (Ninov et al., 2007), thus forming a new epidermal sheet and cuticle. After eclosion, the adult epidermal cells (AECs) are substantially smaller than their larval counterparts and are more rounded in shape (Scherfer et al., 2013). Like their larval counterparts (Galko and Krasnow, 2004), they are capable of undertaking physiological responses such as wound healing (Losick et al., 2013; Ramet et al., 2002). Adult flies undergo a genetically programmed aging process in which the total average lifespan can be shortened by certain mutations (Juhasz et al., 2007; Muñoz-Alarcón et al., 2007) and lengthened by others (Clancy et al., 2001; Libert et al., 2007). In a striking example of a tissue-specific aging program, many of the AECs grow thinner, lose the membranes intervening between nuclei, and eventually lose nuclei too as adult flies age (Scherfer et al., 2013).

The question of whether larval stages can undergo a normal ‘aging’ process, either during the normal window of development or during a prolonged version of this window, has not, to our knowledge, been addressed in the era of molecular/genetic aging research. We approached this question experimentally in two distinct ways. First, we manipulated the larval diet to create ‘longer-lived’ larvae. We did this in control larvae, and in mutants that would normally accelerate or decelerate aging at the adult stage (Juhasz et al., 2007; Muñoz-Alarcón et al., 2007). We then examined the larval barrier epidermis for the normal morphological hallmarks of adult aging (Scherfer et al., 2013) – primarily, loss of membranes intervening between nuclei. Second, we developed a protocol that effects a ‘heterochronic’ persistence of LECs into the adult epidermal sheet. We then examined whether these hybrid epidermal sheets containing both LECs and AECs, and the different cell types within them, underwent a normal process of adult skin aging. The results are presented and discussed below.

## MATERIALS AND METHODS

### Fly stocks and aging

*Drosophila* were reared at 25°C on standard cornmeal medium under a 12 h:12 h light:dark cycle. Aging experiments using adult flies were performed as described in Scherfer et al., 2013. Virgin females were collected and maintained on fly food for the indicated number of days.

For most experiments, *w^1118^* was used as a control strain. Short-lived *lam^G262^* mutants (Morin et al., 2001), which have accelerated epidermal aging at the adult stage, and short-lived *Atg7^d77^* mutants (Juhasz et al., 2007), which have decelerated epidermal aging in adults (Scherfer et al., 2013) were used to examine aging in larval epidermis. The *A29-GAL4* larval/adult epidermal driver was isolated in a screen for Gal4 insertions expressed in the larval epidermis and was combined with *UAS-DsRed2Nuc8* (Lesch et al., 2010) to label epidermal cell nuclei (*A29>DsRed2Nuc8*). A29>*DsRed2Nuc8* was crossed with *w^1118^* to serve as a control. *lam^G262^* mutants and *Atg7^d77^* mutants were combined with *A29>DsRed2Nuc8*, respectively, and were crossed to themselves to create a mutant background with epidermal cell nuclei labeled. *A58-Gal4* (Galko and Krasnow, 2004) and *e22c-Gal4* (Lawrence et al., 1995) label LECs and larval epidermal histoblasts. The *Escargot-Gal4* driver (*Esg-Gal4*) (Hayashi et al., 2002) crossed to a line carrying a UAS-flp out cassette (Evans et al., 2009) and a Stinger fluorescence transgene (eGFP with a nuclear tag) under the control of the ubiquitin promoter (*w; UAS-Flp; Ubi-p63E[FRT.STOP]Stinger*, Bloomington stock 28282) was used to label the nuclei of larval epidermal histoblasts and the descendants of these cells at the adult stage.

### Synthesis and use of E2M

The *erg2Δ::TRP1* yeast was a kind gift from Dr Renato Paro (University of Basel, Switzerland). Culture medium with *erg2Δ::TRP1* yeast (*erg2* medium, E2M) was prepared with a modified version of the protocol described by Katsuyama and Paro (2013). The yeast cells were harvested and heat inactivated at 85°C for 5 min. E2M is composed of 7.5% heat-inactivated *erg2Δ::TRP1* yeast paste, 1.0% glucose and 1.2% agar solution. Penicillin/streptomycin was added as an antibacterial agent. Flies were grown on media containing either brewer's yeast (NM) or E2M for 1 day at 25°C to lay eggs and the parent flies were removed. Hatched larvae were allowed to develop on NM or E2M, and their epidermis was analyzed at the indicated days.

### UV irradiation

Mid-L3 larvae anesthetized with ether were mounted on microscope slides so that the lateral sides were exposed to UV. The glass slides with larvae were placed in a Spectrolinker XL-1000 ultraviolet crosslinker (Spectronics Corporation). The intensity of UV irradiation (mJ cm^−2^) was measured with an AccuMax UVC reader. A range of UV irradiation from 0 (mock) to 5, 10, 15 and 20 mJ cm^−2^ at a wavelength of 254 nm was used (see Fig. 2C). A range of UV irradiation from 0 (mock) to 4, 7, 11, 12, 14, 15 and 16 mJ cm^−2^ at a wavelength of 254 nm was used on larvae carrying an epidermal Gal4 driver (*A29-Gal4*) and a nuclear-localized red fluorescence transgene (*UAS-dsRed2Nuc*) (see Fig. 3B). The UV irradiation of 15–16 mJ cm^−2^ at a wavelength of 254 nm was used otherwise. The larvae after UV treatment were recovered on fly food at 25°C for further analysis.

### Dissection, immunostaining and microscopy

**Fig. 1.**
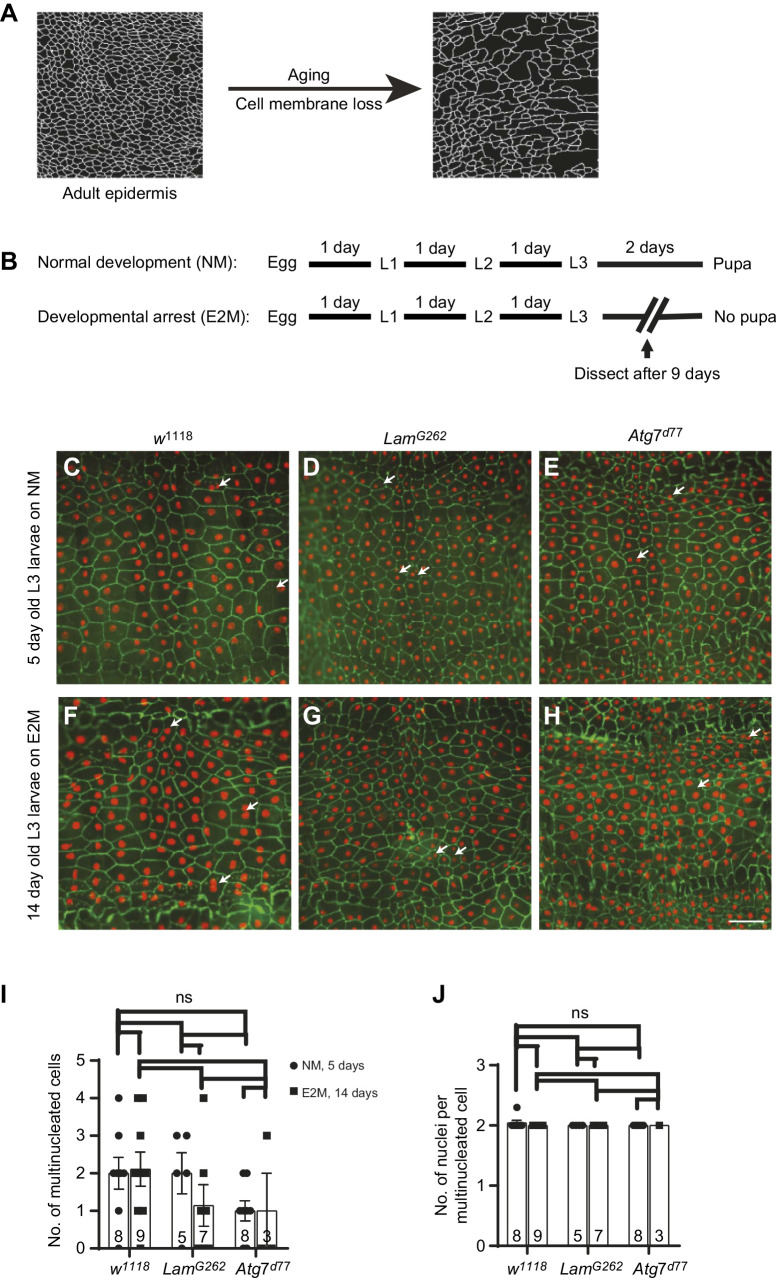
**Longer-lived larvae do not exhibit signs of epidermal aging** (A) Morphological progression of adult epidermal aging. As the adult fly ages, white epidermal membranes are lost, leading to large blank spaces in the epidermal tissue. (B) Schematic diagram of the timing of development on normal media (NM) and *erg2* yeast media (E2M), which causes developmental arrest. (C–H) Dissected epidermal whole-mounts of third instar larvae bearing the indicated mutations and a genetically encoded nuclear label (*A29-Gal4*, *UAS-DsRed2Nuc8*) grown on NM (C–E; 5 day old L3 larvae) or E2M (F–H; 14 day old L3 larvae) and immunostained with anti-Fasciclin III to highlight epidermal cell membranes (green). Epidermal nuclei are labeled in red. Arrows, binucleate larval cells. *w^1118^* control larvae (C,F); *lam^G262^* larvae (D,G); *Atg7^d77^* larvae (E,H). Scale bar (applies to C–H): 100 µm. (I) The mean (±s.e.m.) number of multinucleated cells in a given larval segment as a function of genotype and larval age (5 or 14 days). (J) The mean (±s.e.m.) number of nuclei per multinucleate cell as a function of genotype and larval age. In I and J, the number of individual larval epidermal sheets analyzed is indicated in the respective bars. Two-way ANOVA with Tukey's test was used for statistical analysis. ns, not significant.

**Fig. 2.**
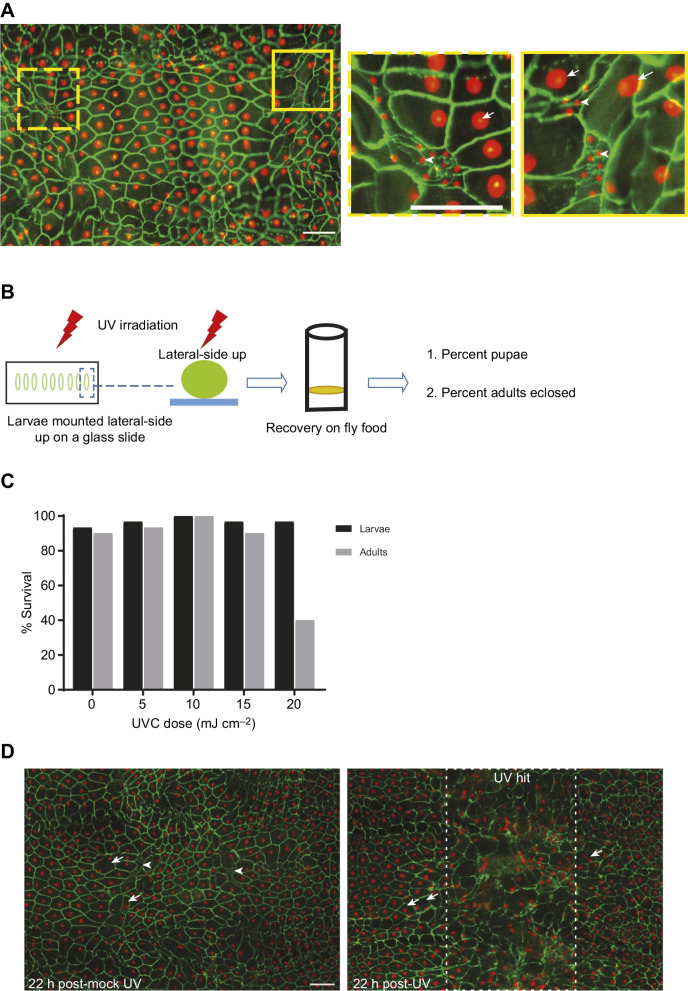
**Irradiation of larval epidermal histoblasts.** (A) Location and morphology of larval epidermal histoblast nests. Dissected epidermal whole-mounts of third instar larvae (*A29-Gal4*, *UAS-dsRed2Nuc8*) immunostained with anti-Fasciclin III to highlight epidermal membranes (green). Nuclei, red. Yellow boxes highlight the epidermal histoblast nests – small clusters of cells that remain diploid within the field of larger endoreplicating larval epidermal cells (LECs). These are shown at higher magnification on the right, indicating the smaller histoblast cells (arrowheads) surrounded by their LEC neighbors (arrows). Scale bars: 100 µm. (B) Schematic diagram of the protocol for UV irradiation and recovery of larvae. (C) Quantification of pupal and adult survival following UV irradiation of third instar larvae with increasing doses of UV (*n*=30). (D) Dissected epidermal whole-mounts of third instar larvae (*A29-Gal4*, *UAS-dsRed2Nuc8*) exposed (right) or mock-exposed (left) to UV and immunostained with anti-Fasciclin III to highlight epidermal membranes (green). Nuclei, red. The white dashed box highlights the epidermal region with disorganized cells induced by UV irradiation. Arrows, binucleated cells. Arrowheads, histoblast nests. Scale bar (applies to both panels): 100 µm.

**Fig. 3.**
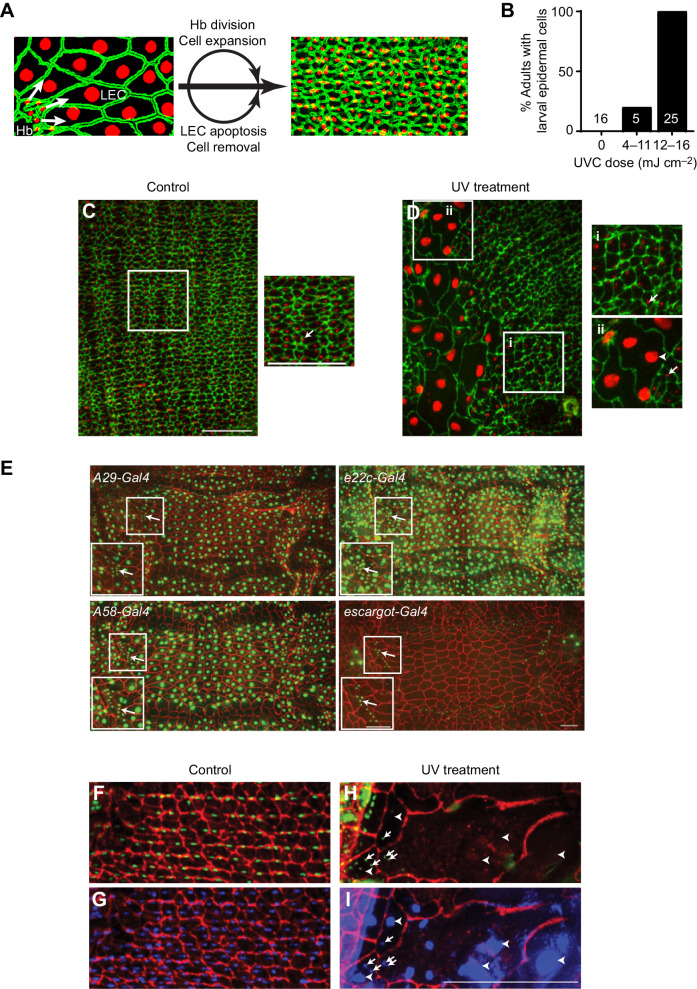
**Persistence of LECs to the adult stage following histoblast irradiation.** (A) Epidermal cell replacement during metamorphosis. At the pupal stage, histoblasts (Hb) proliferate and migrate to replace LECs, which undergo apoptosis and are removed. (B) Quantification of the percentage of adults exhibiting large LEC-like cells as a function of the dose of UV administered during the third larval instar. The number of individual adult epidermal sheets is indicated in the respective bars. (C,D) Dissected epidermal whole-mounts of 1 day old adult abdominal epidermis (*A29-Gal4*, *UAS-dsRed2Nuc8*) immunostained with anti-Fasciclin III (green). Nuclei, red. (C) Panorama of 1 day old control (non-irradiated) epidermal sheet with predominantly small mononuclear cells (before onset of epidermal aging). The white boxed region is shown at higher magnification on the right. Arrow, small AEC nucleus. (D) Panorama of 1 day old irradiated epidermis, showing regions of exclusively small adult epidermal cells (i) and hybrid regions that contain both large LECs and smaller AECs (ii). Arrowhead, large LEC nucleus. Arrows, small AEC nuclei. Scale bars (apply to C and D): 100 µm. (E) Expression of potential lineage trace Gal4 drivers at the larval stage. The respective Gal4 drivers (*A29*, *A58*, *e22c*, *escargot*) are indicated. Each was crossed to *UAS-Flp; Ubi-p63E[FRT.STOP]Stinger* driving a nuclear label (green) and the resulting larvae were dissected and immunostained with anti-Fasciclin III (red). Only *escargot-Gal4* was specific for the epidermal histoblasts (boxed region of small nuclei) without labeling the larger LECs. Arrows, histoblast nests. Scale bars (apply to all panels): 100 µm. (F–I) Lineage tracing of adult epidermal cells using *w; UAS-Flp; Ubi-p63E[FRT.STOP]Stinger.* Progeny of the larval histoblasts in 4 day old control (unirradiated) adults (F,G) were labeled with Stinger (nuclei, green), anti-Fasciclin III (membranes, red) and DAPI (nuclei, blue). Nuclei that are blue without the green label are likely those of the adult body wall muscles that underlie the epidermal sheet. After UV irradiation at the larval stage (H,I), small AEC nuclei of the 4 day old adult were still labeled with both DAPI and Stinger (arrows). Large LEC nuclei were labeled only with DAPI and not the Stinger lineage trace (arrowheads). Scale bar (applies to F–I): 100 µm.

L3 *Drosophila* larvae were dissected and stained as previously described (Burra et al., 2013). Adult flies were anesthetized by briefly exposing them to CO_2_ and dissected as described elsewhere (Scherfer et al., 2013). Briefly, flies were placed on a Sylgard plate (Dow Corning). The head and other appendices were removed using microscissors and the thorax and abdomen were pinned dorsal side up using 0.1 mm diameter dissection needles (Fine Science Tools). After placing the first set of pins, 1× phosphate buffered saline (PBS) was added to the Sylgard plate and any bubbles trapped beneath the ventral abdomen were gently removed. Using dissecting scissors (Fine Science Tools), an incision was made and the flaps were pinned to the side. The viscera and other organs were removed. The dissected samples were fixed in 3.7% formaldehyde prepared in 1× PBS for 1 h and then washed quickly with 1× PBS. After rinsing out the formaldehyde, samples were unpinned and blocked in PHT buffer (phosphate-buffered saline containing 1% heat-inactivated normal goat serum and 0.3% Triton X-100) for 1 h before immunostaining. The samples were incubated overnight in PHT solution containing 1:50 dilution of mouse anti-Fasciclin III (Developmental Studies Hybridoma Bank, 7G10). After primary antibody incubation, the samples were washed with PHT. Next, the samples were incubated overnight in PHT solution containing goat anti-mouse Alexa 647 (Abcam ab150119, 1:500; or Life Technologies A21235, 1:1000). The samples were again washed with 1× PBS containing 0.3% Triton X-100 and mounted in Vectashield mounting medium (Vector Laboratories). For some experiments, adult and persistent LEC nuclei were independently labeled with DAPI at 1:1000 dilution in PHT for 30 min after washes following the secondary antibody incubation. The samples were washed after DAPI incubation and mounted in Vectashield mounting medium. The images in Figs 1, 2A and 3E were captured with a Leica MZ16 FA fluorescence stereomicroscope equipped with a PLAN APO 1.6× stereo-objective and a Jenoptik ProgRes C14 Plus digital camera using Image Pro Plus 7.0 software (Media Cybernetics) or a Hamamatsu ORCA-ER C-4742-80-12AG digital camera using Leica Application Suite X. The other images were captured with an Olympus FV 1000 laser scanning confocal microscope and a UPlanApo 20×/0.70 or UPlanSApo 10×/0.40 objective using FluoView FV10-ASW 3.1 software. Images were later processed with ImageJ and Adobe Photoshop.

### Quantification

**Fig. 4.**
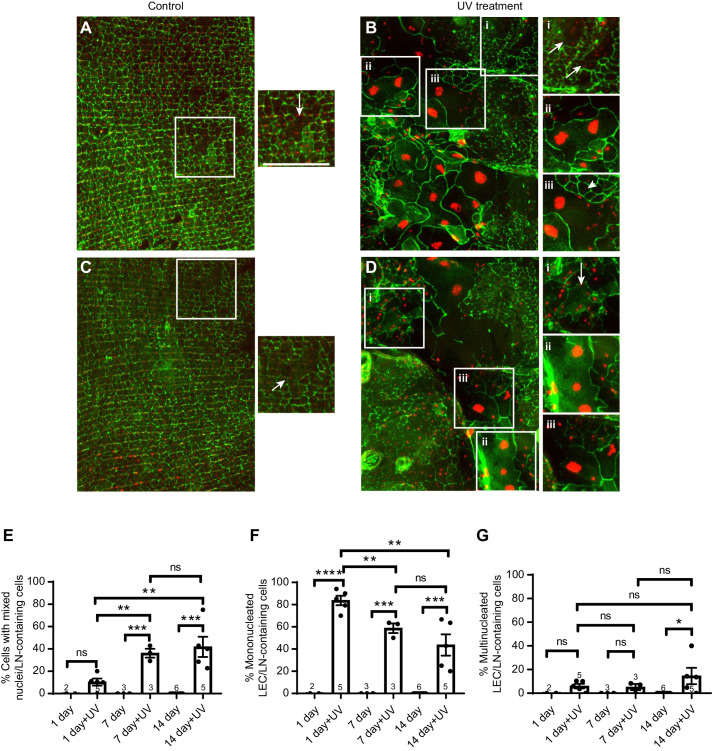
**LECs embedded within the adult epidermis undergo epidermal aging.** (A–D) Dissected whole-mounts of adult abdominal epidermis (*A29-Gal4*, *UAS-dsRed2Nuc8*) immunostained with anti-Fasciclin III (green). Nuclei, red. (A) Panorama of a control (mock-irradiated) 7 day old epidermal sheet where epidermal aging (multinucleate epidermal cells) is now apparent. The white boxed region is shown at higher magnification on the right (arrow, multinucleate cell). (B) The 7 day old irradiated epidermis in panorama exhibited both LECs and AECs. Within the epidermal sheet there were multinucleate cells containing only small adult epidermal nuclei (i, arrows), cells containing multiple large LEC nuclei (ii) and hybrid cells that contained both large LECs and smaller AECs (iii). (C) Panorama of control (mock-irradiated) 14 day old epidermal sheet where epidermal aging (multinucleate epidermal cells) has progressed. At higher magnification (white boxed region), a large AEC with multiple nuclei was observed (arrow). (D) The 14 day old irradiated epidermis in panorama exhibited both LECs and AECs. Within the epidermal sheet there were multinucleate cells containing only small adult epidermal nuclei (i, arrow), cells containing multiple large LEC nuclei (ii) and hybrid cells that contained both large LECs and smaller AECs (iii). Scale bar (applies to all panels): 100 µm. (E–G) Quantification of the mean (s.e.m.) percentage of cells with mixed AEC/LEC nuclei (E), cells with a single LEC nucleus (F), or cells with multiple LEC nuclei but no small AEC nuclei (G) as a function of prior irradiation at the larval stage and indicated adult age. LN, large nucleus. The number of individual adult epidermal sheets analyzed is indicated in the respective bars. Two-tailed unpaired *t*-test was used for statistical analysis for each two groups. ns, not significant; *P<0.05, **P<0.01, ***P<0.001, ****P<0.0001.

In Fig. 1, the number of multinucleated cells and nuclei in each multinucleated cell within a 600 µm×700 µm rectangle (approximately a larval body segment) for each larval whole-mount image was counted. Two-way ANOVA was used for statistical analysis. In Fig. 4, large nuclei were identified using Analyze Particles in ImageJ 1.52. Nuclei channel threshold was set between 50 and 255; particle size was set from 55 µm^2^ to infinity. Large nuclei-containing cells were then counted. The percentage of cells with mixed small (smaller than the low end of the size range for large nuclei) and large nuclei, cells with a single large nucleus, or cells with multiple large nuclei only was calculated. Unpaired *t*-test for each two groups was used for statistical analysis. In Fig. S1, two independent individuals were asked to assign individual epidermal images to one of four classes as defined by the area of epidermal deterioration, as described in Scherfer et al. (2013). Individuals performing the scoring were blind as to genotype/experimental condition. The individual groupings were then averaged.

## RESULTS

### The larval epidermis does not exhibit signs of epidermal aging

Previously, we observed that the *Drosophila* adult epidermis undergoes an age-dependent loss of cell membranes (see Fig. 1A) and nuclei (Scherfer et al., 2013). To help determine whether skin-aging signals are specific to the adult stage, we asked whether larvae, a transitional juvenile form that precedes metamorphosis, exhibit similar age-dependent changes in the epidermis. The *Drosophila* third instar larval stage (L3) normally lasts for 2 days before the puparial molt. The time spent in this stage can be substantially prolonged through nutrient deprivation that precludes synthesis of the molting hormone (Parkin and Burnet, 1986). Larvae grown on media containing yeast mutant for the *erg2* gene cannot synthesize molting hormone and do not pupariate (Katsuyama and Paro, 2013). We developed a scheme to grow control larvae (*w^1118^*) and larvae mutant for aging genes on either normal media (NM) or media made with *erg2* mutant yeast (E2M) (Fig. 1B). We introduced into these genetic backgrounds a genetic label for epidermal nuclei (see Materials and Methods) so that we could easily assess multinuclearity. *Drosophila* grown on NM typically reach L3 after 5 days at 25°C and have large polygonal and primarily mononuclear epidermal cells with distinct cell membranes (Fig. 1C). On NM, both *lam^G262^* mutants, which exhibit a short lifespan (Muñoz-Alarcón et al., 2007) and premature/accelerated skin aging as adults (Scherfer et al., 2013), and *Atg7^d77^* mutants (Juhasz et al., 2007), which exhibit decelerated skin aging as adults (Scherfer et al., 2013), exhibited a morphologically normal larval epidermis at the middle of the L3 stage (5 days old; Fig. 1D,E). When grown on E2M, 14 day old L3 larvae of all genotypes tested also showed an epidermal morphology that was indistinguishable from that of the younger larvae grown on NM (Fig. 1F–H).

In control larvae, occasional binucleate cells were observed as previously described (Wang et al., 2015) (Fig. 1I). If epidermal aging similar to that observed in the adult stage were to occur, the number of these cells and the number of nuclei within them would be expected to increase with the prolonged third instar on E2M. This was not observed in control larvae (Fig. 1I,J). Binucleate cells actually decreased in number in *lam^G262^* mutants and were present at lower numbers in *Atg7^d77^* mutants, without decreasing over time (Fig. 1I). The average number of nuclei per multinucleate cell indicated that nearly all multinucleate cells present in larvae were binucleate and this did not change with a longer larval stage (Fig. 1J). Together, these results suggest that there is no equivalent, in larvae, to the progressive deterioration of epidermal cell membranes that is observed in the adult. This is true regardless of whether the larval genotype would or would not exhibit an aging phenotype at the adult stage.

### UV treatment of larval histoblasts to create adults with persistent larval epidermal cells

The adult epidermis is formed through proliferation and migration of larval epidermal histoblasts after the puparial molt (Madhavan and Madhavan, 1980; Ninov et al., 2007). During normal development, these histoblast cells, which are diploid precursors embedded within the polyploid larval epidermis (Fig. 2A), expand and migrate to replace dying LECs. To interfere with this replacement process, and hopefully create *Drosophila* adults that contained persistent LECs, we developed a protocol where the lateral aspect of L3 larvae was irradiated with UV (see Materials and Methods and Fig. 2B). We first defined a UV dose that is not lethal. We observed full survival to the pupal stage up to 20 mJ cm^−2^ of UVC (Fig. 2C). Survival to the adult stage was complete up to 15 mJ cm^−2^ but dropped by over half when the dose was increased to 20 mJ cm^−2^. Observation of the irradiated larval epidermis in a lateral view that allowed us to focus on the histoblast region indicated substantial local tissue disruption compared with control mock-irradiated larvae (Fig. 2D; see also Babcock et al., 2009). The targeted histoblast nests were difficult to pinpoint in irradiated samples and multinucleate cells were present in the irradiated area, as were the usual binucleate cells outside the region of irradiation (Fig. 2D).

Do LECs in irradiated larvae persist through metamorphosis and into the adult stage? To assess this, we irradiated larvae that carried an epidermal Gal4 driver (*A29-Gal4*) and a nuclear-localized red fluorescence transgene (*UAS-dsRed2Nuc*) (Lesch et al., 2010) that allowed us to distinguish between LECs (large cells and nuclei, 1836±639 µm^2^ cell area, *n*=166) and AECs (small cells and nuclei, 138±34 µm^2^ cell area, *n*=77). We hypothesized that targeted UV irradiation might disrupt the normal replacement of LECs by dividing histoblasts during metamorphosis (see Fig. 3A). In the absence of irradiation, no persistent LECs were observed (Fig. 3B) and all of the epidermal cells in the 1 day old adult epidermal monolayer had the small size and faintly labeled small nuclei characteristic of AECs (Fig. 3C). At this early stage after eclosion, the loss of epidermal cell membranes has not yet commenced, multinucleate cells are rare (Scherfer et al., 2013), and no cells with the large size and characteristic large nuclei of LECs (Lesch et al., 2010; Wang et al., 2015) are apparent. At a UV dose of 4–11 mJ cm^−2^, 20% of adults showed presumptive persistent LECs (Fig. 3B). When this dose was in the range 12–16 mJ cm^−2^, still a dose that gives full survival to the adult stage (Fig. 2C), nearly 100% of the adults that eclosed had presumptive persistent LECs embedded within their adult epidermis. The resulting adult epidermal sheets are shown in Fig. 3D. These presumptive persistent LECs (Fig. 3D, arrowhead) were much larger than adjacent AECs (Fig. 3D, arrows), in terms of area, size and brightness of the nucleus. Most of these cells, on day 1, were mononucleate, though some were binucleate or trinucleate (Fig. 3Dii), possibly a result of the prior UV irradiation (Fig. 2D) (Babcock et al., 2009). Procedurally, the technique developed here provides the functional equivalent of a heterochronic transplant of LECs into the adult epidermal sheet, creating adults that possess a hybrid epidermis consisting of both presumptive persistent LECs and resident AECs. The level of LEC multinuclearity in the day one adult samples provides the baseline from which possible aging (increased multinuclearity) can be measured.

To test whether the presumptive persistent LECs were derived from the larval epidermal histoblasts that give rise to abdominal AECs, we performed a lineage trace experiment. We found that the three common larval epidermal drivers (*A58-Gal4*, *A29-Gal4* and *e22c-Gal4*) were expressed in both LECs and epidermal histoblasts at the larval stage (Fig. 3E), making them inappropriate for a lineage trace experiment. However, the *escargot-Gal4* (*Esg-Gal4*) driver, which has been reported to be expressed in a variety of imaginal cells (Ninov et al., 2007; Pitsouli and Perrimon, 2010), was expressed in epidermal histoblasts but not LECs at the larval stage (Fig. 3E), allowing us to trace the progeny of the histoblasts using *UAS-Flp* and an FRT-Stop fluorescent label (see Materials and Methods). Consistent with a previous study (Ninov et al., 2007) we found that AECs, the known progeny of larval epidermal histoblasts, also labeled with the *Esg-Gal4>UAS-Flp; FRT-STOP-Stinger* lineage marker in control adults that had not been irradiated at the larval stage (Fig. 3F). Following UV irradiation, we observed the mixed-nuclei cells described above, which contained both small AEC nuclei and large presumptive LEC nuclei. All of these nuclei stained with DAPI (Fig. 3G,I), an independent label, but only the small AEC nuclei labeled as GFP+ with the *Esg-Gal4>UAS-Flp; FRT-STOP-Stinger* lineage label (Fig. 3H). The lack of labeling of the large presumptive LEC nuclei suggests that they were in fact derived from persistent LECs, as these cells are not lineage derived from the epidermal histoblasts (Ninov et al., 2007). A prior study of *escargot* mutants with aberrant histoblast turnover during metamorphosis found similar large cells in the adult epidermis (Hayashi et al., 1993). Based on these findings and the lineage trace data, we are confident that these large cells are persistent LECs that were not effectively replaced by the proliferating epidermal histoblasts following UV irradiation.

### LECs that persist until the adult stage do undergo epidermal aging

We next asked what happens to these persistent LECs and the resident AECs as the adults with a hybrid epidermal sheet age. Importantly, adults that had been irradiated at the larval stage appeared to undergo a normal aging process compared with unirradiated controls (Fig. S1) when we examined the adult epidermal sheet in a region that did not contain persistent LECs. We reasoned that if LECs are immune to adult epidermal aging signals, as might be suggested by their behavior in longer-lived larvae, then these cells would stay primarily mononucleate and not increase in multinuclearity as the adult ages. In contrast, if LECs can respond to epidermal aging signals when embedded in the adult epidermis, then we would expect them to lose membranes both between themselves and with the neighboring AECs, resulting in an increase in multinuclearity. The latter is what we observed. In the 7 day old mock-irradiated adult epidermis (Fig. 4A), AECs began to lose some of the membranes intervening between the cells, resulting in the previously observed skin aging phenotype: multinucleate cells (Fig. 4A, arrow). We observed three types of multinucleate cells following irradiation in the 7 day old hybrid epidermis (Fig. 4B). First (Fig. 4Bi, arrows), we saw the ‘standard’ adult epidermal aging phenotype: multinucleate cells consisting solely of small AEC nuclei. Second (Fig. 4Bii), we saw multinucleate cells that contained two (or sometimes more) large LEC nuclei. Third, and most tellingly, we observed multinucleate cells that contained both large LEC nuclei and small AEC nuclei (Fig. 4Biii shows a section of a cell that contains one large LEC nucleus and 9 smaller AEC nuclei, just within the highlighted region). When we quantified the incidence of these mixed nuclei cells, we found that at day 1 of adulthood they were not present in mock-irradiated individuals and were present at low numbers following irradiation (Fig. 4E). The incidence of these mixed nuclei cells increased significantly as the irradiated adults aged to 7 days (Fig. 4E), concomitant with a decrease in the incidence of mononucleate LECs with increasing age (Fig. 4F). Multinucleation of cells that contained only LEC nuclei (no AEC nuclei present) did not change appreciably with age, except from 7 to 14 days (Fig. 4G).

The same diversity of cell types was present as the adults aged to 14 days. In the absence of irradiation, the loss of epidermal membranes progressed with age (compare Fig. 4A with C, arrows). With irradiation, the same diverse cell hybrid cell types seen at 7 days (Fig. 4Biii) were present at 14 days (Fig. 4Diii). A slight, but not significant increase in mixed nuclei cells was observed from 7 days to 14 days of adult age (Fig. 4E). The presence of these various cell types and in particular the increased incidence of mixed nuclei cells over time is strong evidence that LECs heterochronically persistent in the adult epidermal sheet can respond to epidermal aging signals with the same cellular behavior (loss of membranes intervening between nuclei) that is exhibited by AECs during epidermal aging.

## DISCUSSION

Classical experiments in *Drosophila* and other insects have explored various aspects of whether prolonging transitional larval development stages can impact the progression of later stages of development. For instance, prolonging the larval stage in *Drosophila ampelophila* by nutrient deprivation does not lengthen the subsequent duration of metamorphosis (Northrop, 1917). Similarly, prolonging larval life in *Drosophila melanogaster* by lowering the temperature does not have a substantial effect on the duration of the subsequent adult stage (Alpatov and Pearl, 1929). In beetles (*Trogoderma glabrum*), food deprivation leads to a ‘retrogressive’ molting where the new larvae are smaller than the previous stage. Interestingly, these larvae show physiological symptoms of senescence (increased ploidy) primarily in the fat body (Beck and Bharadwaj, 1972). Several studies have examined age-related cellular changes in the adult gut (Miquel et al., 1972), skeletal muscle (Demontis and Perrimon, 2010) and barrier epidermis (Scherfer et al., 2013), but creating adults where larval cells persist has largely been limited to examining the regenerative capacity of imaginal discs (Bryant, 1975), structures that do not persist into the adult in their larval pattern/form. Here, we sought to develop experimental paradigms that would allow us to examine ‘aging’ in a long-lived larval stage and in larval cells heterochronically persisting into the adult stage.

Our results suggest that LECs, when held in a prolonged larval stage, do not undergo a morphological aging process comparable to that of adults. During the normal span of larval development, LECs do not show any of the hallmarks of ‘adult’ aging: loss of membranes between nuclei leading to syncytia or loss of nuclei themselves (Scherfer et al., 2013). Even if the larval stage is prolonged, LECs still do not show any of the signs of age-dependent morphological changes that have been defined in the adult. One might expect *lamin* mutant larvae (Muñoz-Alarcón et al., 2007), which exhibit accelerated epidermal aging in adults (Scherfer et al., 2013), to possibly show signs of epidermal aging at the larval stage. This was not observed, even though the time window examined (days) is sufficient at the adult stage for AECs to have an appearance of being weeks old. LEC morphology was also unchanged in autophagy mutants (Juhasz et al., 2007) that delay epidermal aging at the adult stage (Scherfer et al., 2013) even though their adult lifespan is shortened.

There are several possible explanations for the inability of LECs to show morphological signs of aging, even when the larval stage is prolonged. One is that there is a chemical or physical signal in larvae that inhibits aging. This could be juvenile hormone (Riddiford et al., 2010) or perhaps a mechanical property such as larval turgor pressure. A second possibility is that larvae simply do not produce the systemic signal(s) that might accompany normal adult aging. A variant of this idea is that these signals exist, but that LECs are not responsive to them at this stage. Our heterochronic transplantation experiment tested whether persistent LECs can respond to aging signals present in the adult. The identification of the large cells we observed in the adult epidermis as LECs is supported by prior studies of escargot escaper adult (Hayashi et al., 1993) lineage tracing of epidermal histoblast progeny. Importantly, we were able to examine the cellular morphology of persistent LECs in a tissue that normally undergoes an age-related morphological progression. The resulting epidermis, a hybrid of LECs and AECs, underwent a normal adult epidermal aging program in which all cells participated. This suggests that LECs do have the capacity to respond to systemic aging signals present in the adult or have been released from inhibition of aging by lack of exposure to juvenile signals.

Most adult insect tissues are epithelial in nature and are not, as in vertebrates, replenished during adult life by resident stem cells. Each of these tissues has its own organ-specific aging program that can likely be monitored at the cellular level. For those tissues, such as the barrier epidermis, which have a contribution from nests of imaginal cells that are set aside at the larval stage, one can imagine using a similar irradiation strategy to interfere with replacement of the larval cells during metamorphosis. Another such tissue is the *Drosophila* tracheal system (Weaver and Krasnow, 2008). Although the organ-specific aging program of this tissue has not been examined in cellular detail, some details of the replacement process are known, including its dependence on FGF signaling (Chen and Krasnow, 2014), suggesting that either irradiation-based or genetic strategies (interfering with FGF signaling) might be viable strategies for creating heterochronic animals with a mixture of larval and adult tracheal cells. We hope that the experimental strategies outlined here will prove adaptable to other tissues, allowing an examination of how generalizable the result obtained here, with barrier epidermal cells, proves to be.

## Supplementary Material

Supplementary information
