# Supplementary material for: Drosophila larval epidermal cells only exhibit epidermal aging when they persist to the adult stage
Source: J Exp Biol. 2021 May 6;224(9):jeb240986. doi: 10.1242/jeb.240986 (PMC8126450; doi:10.1242/jeb.240986)
Supplement: Supplementary information [file jexbio-224-240986-s1.pdf]

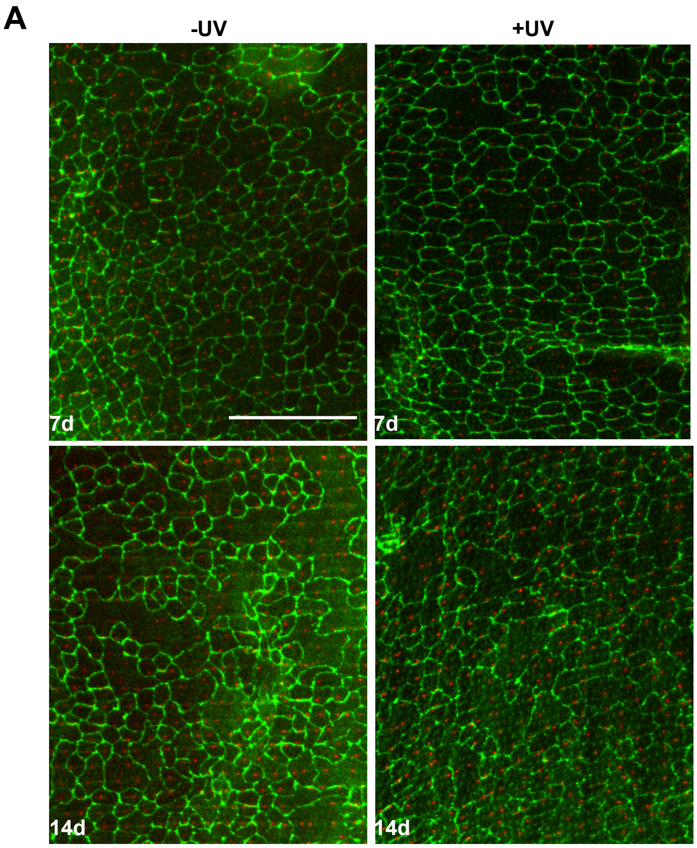

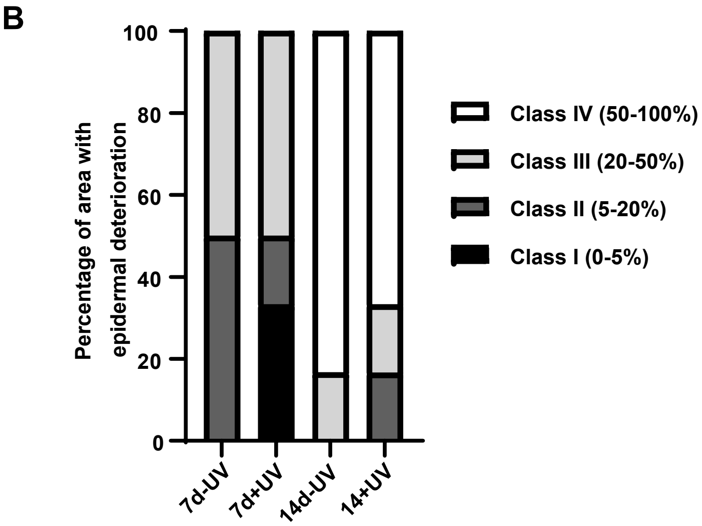

Figure S1. The adult epidermis UV-irradiated at the larval stage still undergoes epidermal aging

(A) Dissected whole mounts of adult abdominal epidermis (*A29-Gal4, UAS-dsRed2Nuc8*) immunostained with anti-Fasciclin III (green). Nuclei, red. Scale bar, 100 µm. (B). Quantification of epidermal deterioration using a semi-quantitative metric (see methods). N (individual adult epidermal sheets analyzed)=3. All comparisons between different groups were significantly different using the Chi square test ($p < 0.0001$).

Journal of Experimental Biology • Supplementary information
